# Methyl (*E*)-2-[(3-chloro-4-cyano­phenyl)­imino]-4-(4-chloro­phen­yl)-6-methyl-1,2,3,4-tetra­hydro­pyrimidine-5-carboxyl­ate

**DOI:** 10.1107/S1600536812039451

**Published:** 2012-09-22

**Authors:** K. N. Venugopala, Susanta K. Nayak, Bharti Odhav

**Affiliations:** a Department of Biotechnology and Food Technology, Durban University of Technology, Durban 4001, South Africa; b Center for Nano Science and Technology @ Polimi, Istituto Italiano di Tecnologia, Via Pascoli 70/3, 20133 Milan, Italy

## Abstract

In the title compound, C_20_H_16_Cl_2_N_4_O_2_, the dihedral angles between the planes of the chloro­phenyl, chloro­cyano­phenyl­imine and ester groups and the plane of the six-membered tetra­hydro­pyrimidine ring are 86.9 (2), 72.6 (2) and 7.9 (2)°, respectively. The Cl atom substituent on the cyano­phenyl ring is disordered over two rotationally related sites [occupancy factors 0.887 (2):0.113 (2)], while the mol­ecular conformation is stabilized by the presence of an intra­molecular aromatic C—H⋯π inter­action. Both N—H groups participate in separate inter­molecular hydrogen-bonding associations with centrosymmetric cyclic motifs [graph sets *R*_2_^2^(8) and *R*_2_^2^(12)], resulting in ribbons parallel to [010]. Further weak C—H⋯O hydrogen bonds link these ribbons into a two-dimensional mol­ecular assembly.

## Related literature

For crystal structures of the dihydro­pyrimidines, see: Nayak *et al.* (2010 ▶); Nayak, Venugopala, Govender *et al.* (2011 ▶); Nayak, Venugopala, Chopra & Guru Row (2011 ▶). For background on the applications of dihydro­pyrimidines, see: Kappe (2000 ▶). For graph-set analysis, see: Bernstein *et al.* (1995 ▶).
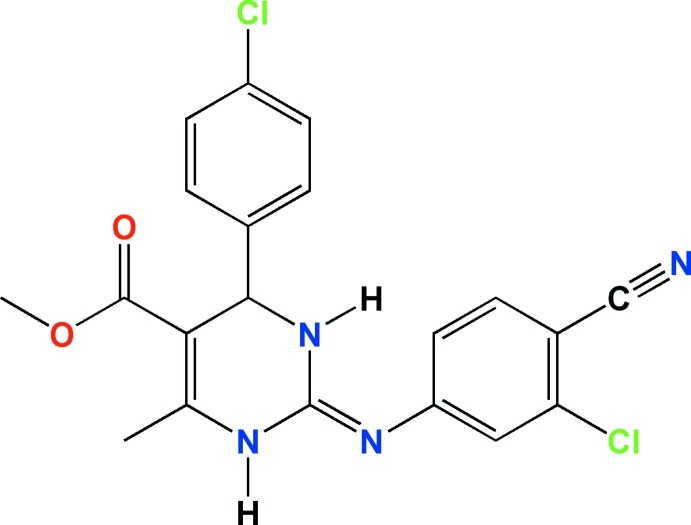


## Experimental

### 

#### Crystal data

C_20_H_16_Cl_2_N_4_O_2_
*M* *_r_* = 415.27
Monoclinic, 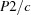

*a* = 11.905 (8) Å
*b* = 13.729 (9) Å
*c* = 12.782 (8) Å
β = 108.366 (14)°
*V* = 1983 (2) Å^3^
*Z* = 4
Mo *K*α radiation
μ = 0.35 mm^−1^
*T* = 173 K
0.23 × 0.12 × 0.03 mm

#### Data collection

Bruker Kappa DUO APEXII diffractometer
Absorption correction: multi-scan (*SADABS*; Bruker, 2008 ▶) *T*_min_ = 0.924, *T*_max_ = 0.990
9454 measured reflections
3497 independent reflections
2324 reflections with *I* > 2σ(*I*)
*R*_int_ = 0.029
Standard reflections: 0

#### Refinement

*R*[*F* ^2^ > 2σ(*F* ^2^)] = 0.046
*wR*(*F* ^2^) = 0.130
*S* = 1.01
3497 reflections
259 parameters
H-atom parameters constrained
Δρ_max_ = 0.41 e Å^−3^
Δρ_min_ = −0.37 e Å^−3^

### 

Data collection: *APEX2* (Bruker, 2008 ▶); cell refinement: *SAINT* (Bruker, 2008 ▶); data reduction: *SAINT*; program(s) used to solve structure: *SHELXS97* (Sheldrick, 2008 ▶); program(s) used to refine structure: *SHELXL97* (Sheldrick, 2008 ▶); molecular graphics: *ORTEP-3 for Windows* (Farrugia, 1997 ▶) and *Mercury* (Macrae *et al.*, 2008 ▶); software used to prepare material for publication: *PLATON* (Spek, 2009 ▶) and *PARST* (Nardelli, 1995 ▶).

## Supplementary Material

Crystal structure: contains datablock(s) global, I. DOI: 10.1107/S1600536812039451/zs2233sup1.cif

Structure factors: contains datablock(s) I. DOI: 10.1107/S1600536812039451/zs2233Isup2.hkl

Supplementary material file. DOI: 10.1107/S1600536812039451/zs2233Isup3.cml

Additional supplementary materials:  crystallographic information; 3D view; checkCIF report

## Figures and Tables

**Table 1:** Hydrogen-bond geometry (Å, °) *Cg*1 is the mid-point of the C3=C4 bond.

*D*—H⋯*A*	*D*—H	H⋯*A*	*D*⋯*A*	*D*—H⋯*A*
N1—H1⋯N4*A* ^i^	0.88	2.21	2.981 (4)	147
N2—H2⋯N3^ii^	0.88	2.09	2.966 (4)	172
C15*A*—H15*A*⋯O1^iii^	0.95	2.39	3.322 (4)	169
C12—H12⋯*Cg*1	0.95	2.85	3.290 (2)	109

Symmetry codes: (i) 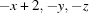; (ii) 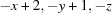; (iii) 
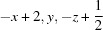
.

**Fig. 1.**
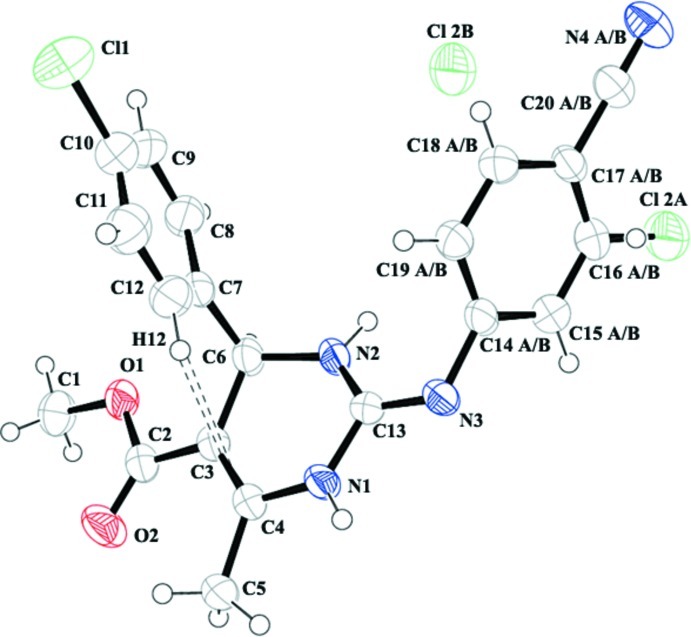
A view of the title compound with the atom numbering scheme and displacement ellipsoids for non-H atoms drawn at the 50% probability level. The intramolecular C—H···π interaction is shown as dashed lines. The disordered chlorine positions are differentiated as A and B.

**Fig. 2.**
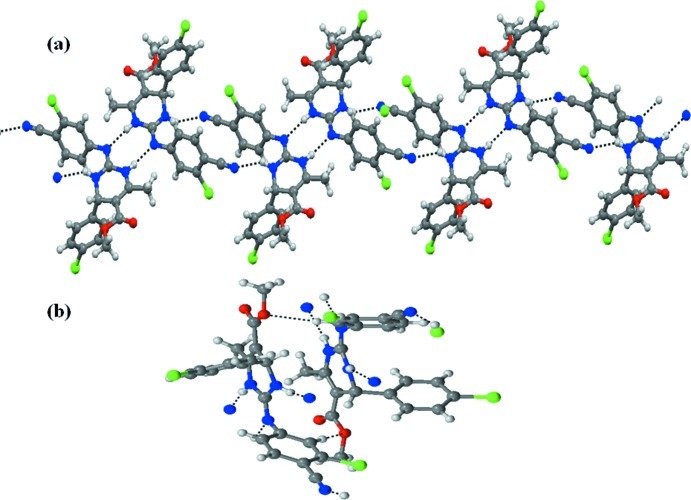
(a) Intermolecular N—H···N hydrogen-bonding associations form an infinite ribbon structure. (b) Further C—H···O hydrogen bonds link the ribbons giving a two-dimensional network structure.

## supplementary crystallographic information

### Comment

The multifunctionalized dihydropyrimidones (DHPMs) are prime target molecules for their therapeutic and pharmacological properties (Kappe, 2000). Due to the vast range of applications of this class of compounds we have been investigating conformational and packing features of tetrahydropyrimidine derivatives of this title compound (Nayak *et al.*, 2010; Nayak, Venugopala, Govender *et al.*, 2011; Nayak, Venugopala, Chopra & Guru Row (2011). In a continuation of our work on synthesis of heterocyclic compounds for biological properties, herein we report the single-crystal structure of the title compound, C_20_H_16_Cl_2_N_4_O_2_.

In this molecule (Fig. 1), the dihedral angles between the planes of the 4-chlorophenyl, 3-chloro-4-cyanophenylimino and ester groups (O2/C2/O1/C1) and the plane of the six-membered tetrahydropyrimidine ring are 86.9 (2)°, 72.6 (2)° and 7.9 (2)° respectively. The conformation of the molecule is stabilized by an intra-molecular C—H···π interaction (Table 1) wherein the aryl hydrogen H12 is oriented towards the π electrons of the C3═C4 bond. The *meta*-related chlorine substituent on the cyanophenyl ring is disordered over two rotationally-related sites [occupancy factors 0.887 (2) (*A*): 0.113 (2) *B*]. Both N—H groups participate in separate intermolecular hydrogen-bonding associations giving centrosymmetric cyclic motifs [graph sets *R*_2_^2^(8) and *R*_2_^2^(12) (Bernstein *et al.*, 1995)], resulting in ribbons parallel to [010] (Fig. 2*a*). Further weak C—H···O hydrogen bonds (Fig. 2*b*) link these ribbons into a two-dimensional molecular assembly. Present also is a short intermolecular Cl···Cl interaction [Cl1···Cl2*B*^iv^; 2.884 (7) Å (symmetry code -*x* + 1, *y*, -*z* - 1/2)].

### Experimental

A mixture of methyl-2-chloro-4-(*p*-chlorophenyl)-6-methyl-1,4-dihydropyrimidine-5-carboxylate (1 mmol), 4-amino-2-chlorobenzonitrile (1 mmol) and methanamine (1 mmol) in 2-propanol (5 ml) was refluxed for 10 h. The reaction completion was monitored by TLC. The reaction medium was cooled to room temperature, the product was filtered, washed with cold 2-propanol and dried to obtain the crude product. The product was purified by recrystallization using ethanol in 69% yield as a yellow solid (m.p. 431 (2) K). Crystals suitable for single-crystal X-ray study were obtained from methanol solvent using slow evaporation at room temperature.

### Refinement

The 3-chloro-4-cyanophenylimino group was treated as disordered over two possible rotation-related sites (*A* and *B*), having refined site occupancy factors of 0.887 (2) and 0.113 (2), respectively. All H atoms were positioned geometrically with N—H = 0.88 Å, C—H = 0.95–1.00 Å and refined using a riding model with *U*_iso_(H) = 1.2*U*_eq_(C/N) except for the methyl group where *U*_iso_(H) = 1.5*U*_eq_(C).

### Crystal data

**Table:**

C_20_H_16_Cl_2_N_4_O_2_	*F*(000) = 856
*M**_r_* = 415.27	*D*_x_ = 1.391 Mg m^−^^3^
Monoclinic, *P*2/*c*	Melting point: 431(2) K
Hall symbol: -P 2yc	Mo *K*α radiation, λ = 0.71073 Å
*a* = 11.905 (8) Å	Cell parameters from 650 reflections
*b* = 13.729 (9) Å	θ = 1.5–25.0°
*c* = 12.782 (8) Å	µ = 0.35 mm^−^^1^
β = 108.366 (14)°	*T* = 173 K
*V* = 1983 (2) Å^3^	Plate, yellow
*Z* = 4	0.23 × 0.12 × 0.03 mm

### Data collection

**Table:**

Bruker Kappa DUO APEXII diffractometer	3497 independent reflections
Radiation source: fine-focus sealed tube	2324 reflections with *I* > 2σ(*I*)
Graphite monochromator	*R*_int_ = 0.029
0.5° φ scans and ω scans	θ_max_ = 25.0°, θ_min_ = 1.5°
Absorption correction: multi-scan (*SADABS*; Bruker, 2008)	*h* = −14→13
*T*_min_ = 0.924, *T*_max_ = 0.990	*k* = −16→16
9454 measured reflections	*l* = −7→15

### Refinement

**Table:**

Refinement on *F*^2^	Primary atom site location: structure-invariant direct methods
Least-squares matrix: full	Secondary atom site location: difference Fourier map
*R*[*F*^2^ > 2σ(*F*^2^)] = 0.046	Hydrogen site location: inferred from neighbouring sites
*wR*(*F*^2^) = 0.130	H-atom parameters constrained
*S* = 1.01	*w* = 1/[σ^2^(*F*_o_^2^) + (0.0628*P*)^2^ + 0.7735*P*] where *P* = (*F*_o_^2^ + 2*F*_c_^2^)/3
3497 reflections	(Δ/σ)_max_ = 0.001
259 parameters	Δρ_max_ = 0.41 e Å^−^^3^
0 restraints	Δρ_min_ = −0.37 e Å^−^^3^

### Special details

**Table:**

Geometry. All s.u.'s (except the s.u. in the dihedral angle between two l.s. planes) are estimated using the full covariance matrix. The cell s.u.'s are taken into account individually in the estimation of s.u.'s in distances, angles and torsion angles; correlations between s.u.'s in cell parameters are only used when they are defined by crystal symmetry. An approximate (isotropic) treatment of cell s.u.'s is used for estimating s.u.'s involving l.s. planes.
Refinement. Refinement of *F*^2^ against ALL reflections. The weighted *R*-factor *wR* and goodness of fit *S* are based on *F*^2^, conventional *R*-factors *R* are based on *F*, with *F* set to zero for negative *F*^2^. The threshold expression of *F*^2^ > 2σ(*F*^2^) is used only for calculating *R*-factors(gt) *etc*. and is not relevant to the choice of reflections for refinement. *R*-factors based on *F*^2^ are statistically about twice as large as those based on *F*, and *R*-factors based on ALL data will be even larger.

### Fractional atomic coordinates and isotropic or equivalent isotropic displacement parameters (Å^2^)

**Table:**

	*x*	*y*	*z*	*U*_iso_*/*U*_eq_	Occ. (<1)
Cl2A	1.27478 (8)	0.07230 (7)	0.07772 (9)	0.0732 (3)	0.8872 (16)
C14A	1.0144 (2)	0.26844 (18)	−0.0167 (2)	0.0429 (7)	0.8872 (16)
C15A	1.1235 (3)	0.2245 (2)	0.0335 (2)	0.0511 (7)	0.8872 (16)
H15A	1.1837	0.2590	0.0874	0.061*	0.8872 (16)
C16A	1.1443 (2)	0.1307 (2)	0.0047 (2)	0.0490 (7)	0.8872 (16)
C17A	1.0592 (2)	0.07963 (17)	−0.0761 (2)	0.0387 (6)	0.8872 (16)
C18A	0.9496 (2)	0.12388 (18)	−0.1256 (2)	0.0424 (6)	0.8872 (16)
H18A	0.8900	0.0898	−0.1806	0.051*	0.8872 (16)
C19A	0.9270 (2)	0.21625 (19)	−0.0957 (2)	0.0435 (6)	0.8872 (16)
H19A	0.8514	0.2448	−0.1291	0.052*	0.8872 (16)
C20A	1.0824 (3)	−0.0174 (2)	−0.1067 (2)	0.0449 (7)	0.8872 (16)
N4A	1.0998 (2)	−0.09457 (18)	−0.1320 (2)	0.0584 (7)	0.8872 (16)
Cl2B	0.8417 (6)	0.0473 (6)	−0.1826 (7)	0.0732 (3)	0.1128 (16)
C14B	1.0144 (2)	0.26844 (18)	−0.0167 (2)	0.0429 (7)	0.1128 (16)
C15B	1.1235 (3)	0.2245 (2)	0.0335 (2)	0.0511 (7)	0.1128 (16)
H15B	1.1837	0.2590	0.0874	0.061*	0.1128 (16)
C16B	1.1443 (2)	0.1307 (2)	0.0047 (2)	0.0490 (7)	0.1128 (16)
H16B	1.2182	0.1007	0.0410	0.059*	0.1128 (16)
C17B	1.0592 (2)	0.07963 (17)	−0.0761 (2)	0.0387 (6)	0.1128 (16)
C18B	0.9496 (2)	0.12388 (18)	−0.1256 (2)	0.0424 (6)	0.1128 (16)
C19B	0.9270 (2)	0.21625 (19)	−0.0957 (2)	0.0435 (6)	0.1128 (16)
H19B	0.8514	0.2448	−0.1291	0.052*	0.1128 (16)
C20B	1.0824 (3)	−0.0174 (2)	−0.1067 (2)	0.0449 (7)	0.1128 (16)
N4B	1.0998 (2)	−0.09457 (18)	−0.1320 (2)	0.0584 (7)	0.1128 (16)
Cl1	0.35886 (9)	0.15552 (9)	−0.18125 (10)	0.1015 (4)	
O1	0.67082 (15)	0.36934 (13)	0.30483 (14)	0.0440 (5)	
O2	0.6947 (2)	0.53124 (14)	0.30330 (18)	0.0635 (6)	
N1	0.88569 (18)	0.31127 (15)	0.12045 (18)	0.0418 (5)	
H1	0.9209	0.2541	0.1288	0.050*	
N2	0.89937 (19)	0.47517 (14)	0.08817 (18)	0.0445 (6)	
H2	0.9239	0.5226	0.0544	0.053*	
N3	0.9962 (2)	0.36677 (15)	0.0096 (2)	0.0480 (6)	
C1	0.6064 (3)	0.3833 (2)	0.3825 (2)	0.0545 (8)	
H1A	0.6603	0.4078	0.4523	0.082*	
H1B	0.5725	0.3211	0.3952	0.082*	
H1C	0.5426	0.4305	0.3525	0.082*	
C2	0.7139 (2)	0.4517 (2)	0.2724 (2)	0.0429 (6)	
C3	0.7800 (2)	0.42850 (18)	0.1967 (2)	0.0393 (6)	
C4	0.8352 (2)	0.49915 (19)	0.1574 (2)	0.0405 (6)	
C5	0.8350 (3)	0.60622 (18)	0.1828 (2)	0.0478 (7)	
H5A	0.7538	0.6311	0.1557	0.072*	
H5B	0.8843	0.6411	0.1466	0.072*	
H5C	0.8667	0.6161	0.2627	0.072*	
C6	0.7861 (2)	0.32283 (18)	0.1639 (2)	0.0398 (6)	
H6	0.8027	0.2821	0.2320	0.048*	
C7	0.6733 (2)	0.28406 (18)	0.0795 (2)	0.0403 (6)	
C8	0.6315 (3)	0.1926 (2)	0.0943 (3)	0.0552 (8)	
H8	0.6691	0.1570	0.1597	0.066*	
C9	0.5350 (3)	0.1527 (2)	0.0139 (3)	0.0697 (10)	
H9	0.5076	0.0894	0.0234	0.084*	
C10	0.4795 (3)	0.2057 (3)	−0.0795 (3)	0.0625 (9)	
C11	0.5174 (3)	0.2981 (2)	−0.0955 (3)	0.0589 (8)	
H11	0.4778	0.3345	−0.1598	0.071*	
C12	0.6147 (3)	0.3362 (2)	−0.0151 (2)	0.0496 (7)	
H12	0.6420	0.3994	−0.0250	0.060*	
C13	0.9271 (2)	0.38082 (17)	0.0692 (2)	0.0400 (6)	

### Atomic displacement parameters (Å^2^)

**Table:**

	*U*^11^	*U*^22^	*U*^33^	*U*^12^	*U*^13^	*U*^23^
Cl2A	0.0432 (5)	0.0708 (6)	0.0957 (7)	0.0139 (4)	0.0076 (5)	−0.0130 (5)
C14A	0.0484 (16)	0.0313 (14)	0.0634 (18)	−0.0036 (13)	0.0384 (15)	−0.0031 (13)
C15A	0.0442 (16)	0.0458 (16)	0.0676 (19)	−0.0057 (14)	0.0237 (15)	−0.0131 (14)
C16A	0.0393 (15)	0.0455 (16)	0.0652 (18)	0.0060 (13)	0.0207 (14)	−0.0046 (14)
C17A	0.0473 (16)	0.0295 (13)	0.0491 (15)	0.0024 (12)	0.0290 (13)	−0.0010 (12)
C18A	0.0447 (16)	0.0343 (14)	0.0514 (16)	−0.0026 (12)	0.0197 (13)	−0.0027 (12)
C19A	0.0426 (15)	0.0337 (14)	0.0596 (17)	0.0016 (13)	0.0238 (14)	0.0004 (13)
C20A	0.0529 (17)	0.0394 (16)	0.0462 (16)	0.0063 (13)	0.0214 (13)	0.0014 (13)
N4A	0.0752 (18)	0.0419 (15)	0.0620 (16)	0.0152 (13)	0.0273 (14)	−0.0020 (12)
Cl2B	0.0432 (5)	0.0708 (6)	0.0957 (7)	0.0139 (4)	0.0076 (5)	−0.0130 (5)
C14B	0.0484 (16)	0.0313 (14)	0.0634 (18)	−0.0036 (13)	0.0384 (15)	−0.0031 (13)
C15B	0.0442 (16)	0.0458 (16)	0.0676 (19)	−0.0057 (14)	0.0237 (15)	−0.0131 (14)
C16B	0.0393 (15)	0.0455 (16)	0.0652 (18)	0.0060 (13)	0.0207 (14)	−0.0046 (14)
C17B	0.0473 (16)	0.0295 (13)	0.0491 (15)	0.0024 (12)	0.0290 (13)	−0.0010 (12)
C18B	0.0447 (16)	0.0343 (14)	0.0514 (16)	−0.0026 (12)	0.0197 (13)	−0.0027 (12)
C19B	0.0426 (15)	0.0337 (14)	0.0596 (17)	0.0016 (13)	0.0238 (14)	0.0004 (13)
C20B	0.0529 (17)	0.0394 (16)	0.0462 (16)	0.0063 (13)	0.0214 (13)	0.0014 (13)
N4B	0.0752 (18)	0.0419 (15)	0.0620 (16)	0.0152 (13)	0.0273 (14)	−0.0020 (12)
Cl1	0.0658 (6)	0.1042 (8)	0.1193 (8)	−0.0215 (6)	0.0072 (6)	−0.0381 (7)
O1	0.0411 (10)	0.0440 (11)	0.0553 (11)	0.0003 (9)	0.0270 (9)	0.0015 (9)
O2	0.0846 (16)	0.0439 (12)	0.0850 (15)	0.0025 (11)	0.0598 (14)	−0.0096 (11)
N1	0.0393 (12)	0.0288 (11)	0.0661 (14)	0.0043 (9)	0.0293 (11)	−0.0007 (10)
N2	0.0528 (14)	0.0280 (11)	0.0687 (15)	−0.0037 (10)	0.0420 (13)	−0.0065 (10)
N3	0.0545 (14)	0.0296 (11)	0.0752 (16)	−0.0041 (11)	0.0423 (13)	−0.0089 (11)
C1	0.0521 (17)	0.064 (2)	0.0577 (18)	−0.0015 (15)	0.0325 (15)	0.0001 (15)
C2	0.0380 (14)	0.0409 (15)	0.0544 (16)	0.0038 (12)	0.0213 (13)	−0.0015 (13)
C3	0.0371 (14)	0.0342 (14)	0.0518 (16)	0.0005 (12)	0.0214 (12)	−0.0028 (12)
C4	0.0381 (14)	0.0353 (14)	0.0538 (16)	0.0002 (12)	0.0225 (13)	−0.0072 (12)
C5	0.0488 (16)	0.0350 (14)	0.0701 (19)	−0.0037 (13)	0.0339 (15)	−0.0100 (13)
C6	0.0392 (14)	0.0331 (13)	0.0563 (16)	0.0023 (11)	0.0283 (13)	0.0018 (12)
C7	0.0374 (14)	0.0331 (14)	0.0599 (17)	0.0009 (12)	0.0287 (13)	−0.0054 (13)
C8	0.0443 (16)	0.0344 (15)	0.089 (2)	0.0015 (13)	0.0237 (16)	0.0053 (15)
C9	0.0474 (18)	0.0366 (17)	0.123 (3)	−0.0069 (15)	0.024 (2)	−0.0123 (19)
C10	0.0458 (18)	0.061 (2)	0.081 (2)	−0.0060 (17)	0.0200 (17)	−0.0222 (19)
C11	0.0559 (19)	0.071 (2)	0.0545 (18)	−0.0044 (17)	0.0248 (16)	−0.0050 (16)
C12	0.0520 (17)	0.0454 (16)	0.0595 (18)	−0.0070 (14)	0.0290 (15)	−0.0006 (14)
C13	0.0384 (14)	0.0288 (13)	0.0592 (16)	−0.0039 (11)	0.0246 (13)	−0.0074 (12)

### Geometric parameters (Å, º)

**Table:**

Cl2A—C16A	1.736 (3)	N3—C13	1.300 (3)
C14A—C15A	1.392 (4)	C1—H1A	0.9800
C14A—C19A	1.398 (4)	C1—H1B	0.9800
C14A—N3	1.424 (3)	C1—H1C	0.9800
C15A—C16A	1.382 (4)	C2—C3	1.461 (3)
C15A—H15A	0.9500	C3—C4	1.355 (3)
C16A—C17A	1.388 (4)	C3—C6	1.518 (4)
C17A—C18A	1.397 (4)	C4—C5	1.506 (4)
C17A—C20A	1.439 (4)	C5—H5A	0.9800
C18A—C19A	1.375 (4)	C5—H5B	0.9800
C18A—H18A	0.9500	C5—H5C	0.9800
C19A—H19A	0.9500	C6—C7	1.530 (4)
C20A—N4A	1.146 (3)	C6—H6	1.0000
Cl1—C10	1.747 (3)	C7—C8	1.385 (4)
O1—C2	1.359 (3)	C7—C12	1.389 (4)
O1—C1	1.446 (3)	C8—C9	1.389 (4)
O2—C2	1.207 (3)	C8—H8	0.9500
N1—C13	1.337 (3)	C9—C10	1.376 (5)
N1—C6	1.468 (3)	C9—H9	0.9500
N1—H1	0.8800	C10—C11	1.383 (5)
N2—C13	1.377 (3)	C11—C12	1.386 (4)
N2—C4	1.378 (3)	C11—H11	0.9500
N2—H2	0.8800	C12—H12	0.9500
			
C15A—C14A—C19A	119.0 (2)	C2—C3—C6	118.3 (2)
C15A—C14A—N3	119.4 (3)	C3—C4—N2	120.0 (2)
C19A—C14A—N3	121.5 (3)	C3—C4—C5	125.8 (2)
C16A—C15A—C14A	120.0 (3)	N2—C4—C5	114.3 (2)
C16A—C15A—H15A	120.0	C4—C5—H5A	109.5
C14A—C15A—H15A	120.0	C4—C5—H5B	109.5
C15A—C16A—C17A	121.3 (3)	H5A—C5—H5B	109.5
C15A—C16A—Cl2A	119.5 (2)	C4—C5—H5C	109.5
C17A—C16A—Cl2A	119.1 (2)	H5A—C5—H5C	109.5
C16A—C17A—C18A	118.4 (2)	H5B—C5—H5C	109.5
C16A—C17A—C20A	120.9 (3)	N1—C6—C3	108.84 (19)
C18A—C17A—C20A	120.7 (2)	N1—C6—C7	109.2 (2)
C19A—C18A—C17A	120.8 (3)	C3—C6—C7	114.8 (2)
C19A—C18A—H18A	119.6	N1—C6—H6	107.9
C17A—C18A—H18A	119.6	C3—C6—H6	107.9
C18A—C19A—C14A	120.5 (3)	C7—C6—H6	107.9
C18A—C19A—H19A	119.8	C8—C7—C12	118.8 (3)
C14A—C19A—H19A	119.8	C8—C7—C6	119.5 (3)
N4A—C20A—C17A	179.3 (4)	C12—C7—C6	121.6 (2)
C2—O1—C1	115.6 (2)	C7—C8—C9	120.3 (3)
C13—N1—C6	125.1 (2)	C7—C8—H8	119.8
C13—N1—H1	117.5	C9—C8—H8	119.8
C6—N1—H1	117.5	C10—C9—C8	119.5 (3)
C13—N2—C4	123.3 (2)	C10—C9—H9	120.3
C13—N2—H2	118.3	C8—C9—H9	120.3
C4—N2—H2	118.3	C9—C10—C11	121.6 (3)
C13—N3—C14A	116.7 (2)	C9—C10—Cl1	119.7 (3)
O1—C1—H1A	109.5	C11—C10—Cl1	118.8 (3)
O1—C1—H1B	109.5	C10—C11—C12	118.1 (3)
H1A—C1—H1B	109.5	C10—C11—H11	120.9
O1—C1—H1C	109.5	C12—C11—H11	120.9
H1A—C1—H1C	109.5	C11—C12—C7	121.6 (3)
H1B—C1—H1C	109.5	C11—C12—H12	119.2
O2—C2—O1	121.6 (2)	C7—C12—H12	119.2
O2—C2—C3	127.6 (2)	N3—C13—N1	125.5 (2)
O1—C2—C3	110.7 (2)	N3—C13—N2	118.3 (2)
C4—C3—C2	121.0 (2)	N1—C13—N2	116.1 (2)
C4—C3—C6	120.7 (2)		
			
C19A—C14A—C15A—C16A	0.1 (4)	C13—N1—C6—C3	−29.3 (3)
N3—C14A—C15A—C16A	−176.8 (2)	C13—N1—C6—C7	96.7 (3)
C14A—C15A—C16A—C17A	1.9 (4)	C4—C3—C6—N1	18.4 (3)
C14A—C15A—C16A—Cl2A	−173.4 (2)	C2—C3—C6—N1	−161.1 (2)
C15A—C16A—C17A—C18A	−2.3 (4)	C4—C3—C6—C7	−104.4 (3)
Cl2A—C16A—C17A—C18A	173.05 (19)	C2—C3—C6—C7	76.1 (3)
C15A—C16A—C17A—C20A	179.0 (2)	N1—C6—C7—C8	101.1 (3)
Cl2A—C16A—C17A—C20A	−5.7 (4)	C3—C6—C7—C8	−136.3 (2)
C16A—C17A—C18A—C19A	0.6 (4)	N1—C6—C7—C12	−75.4 (3)
C20A—C17A—C18A—C19A	179.4 (2)	C3—C6—C7—C12	47.1 (3)
C17A—C18A—C19A—C14A	1.3 (4)	C12—C7—C8—C9	2.1 (4)
C15A—C14A—C19A—C18A	−1.7 (4)	C6—C7—C8—C9	−174.6 (2)
N3—C14A—C19A—C18A	175.2 (2)	C7—C8—C9—C10	−1.5 (5)
C15A—C14A—N3—C13	−108.4 (3)	C8—C9—C10—C11	0.0 (5)
C19A—C14A—N3—C13	74.8 (3)	C8—C9—C10—Cl1	179.5 (2)
C1—O1—C2—O2	−3.0 (4)	C9—C10—C11—C12	0.8 (5)
C1—O1—C2—C3	178.4 (2)	Cl1—C10—C11—C12	−178.7 (2)
O2—C2—C3—C4	4.3 (5)	C10—C11—C12—C7	−0.2 (4)
O1—C2—C3—C4	−177.2 (2)	C8—C7—C12—C11	−1.2 (4)
O2—C2—C3—C6	−176.2 (3)	C6—C7—C12—C11	175.3 (2)
O1—C2—C3—C6	2.3 (3)	C14A—N3—C13—N1	10.1 (4)
C2—C3—C4—N2	178.5 (2)	C14A—N3—C13—N2	−174.5 (2)
C6—C3—C4—N2	−1.0 (4)	C6—N1—C13—N3	−163.9 (3)
C2—C3—C4—C5	−1.0 (4)	C6—N1—C13—N2	20.6 (4)
C6—C3—C4—C5	179.5 (3)	C4—N2—C13—N3	−174.4 (3)
C13—N2—C4—C3	−10.6 (4)	C4—N2—C13—N1	1.4 (4)
C13—N2—C4—C5	168.9 (3)		

### Hydrogen-bond geometry (Å, º)

Cg1 is the midpoint of the C3═C4 bond. [Please check added text]

**Table:**

*D*—H···*A*	*D*—H	H···*A*	*D*···*A*	*D*—H···*A*
N1—H1···N4*A*^i^	0.88	2.21	2.981 (4)	147
N2—H2···N3^ii^	0.88	2.09	2.966 (4)	172
C15*A*—H15*A*···O1^iii^	0.95	2.39	3.322 (4)	169
C12—H12···*Cg*1	0.95	2.85	3.290 (2)	109

Symmetry codes: (i) −*x*+2, −*y*, −*z*; (ii) −*x*+2, −*y*+1, −*z*; (iii) −*x*+2, *y*, −*z*+1/2.

## References

[bb1] Bernstein, J., Davis, R. E., Shimoni, L. & Chang, N.-L. (1995). *Angew. Chem. Int. Ed. Engl.* **34**, 1555–1573.

[bb2] Bruker (2008). *APEX2*, *SAINT* and *SADABS* Bruker AXS Inc., Madison, Wisconsin, USA.

[bb3] Farrugia, L. J. (1997). *J. Appl. Cryst.* **30**, 565.

[bb4] Kappe, C. O. (2000). *Eur. J. Med. Chem.* **35**, 1043–1052. 10.1016/s0223-5234(00)01189-2 11248403

[bb5] Macrae, C. F., Bruno, I. J., Chisholm, J. A., Edgington, P. R., McCabe, P., Pidcock, E., Rodriguez-Monge, L., Taylor, R., van de Streek, J. & Wood, P. A. (2008). *J. Appl. Cryst.* **41**, 466–470.

[bb6] Nardelli, M. (1995). *J. Appl. Cryst.* **28**, 659.

[bb7] Nayak, S. K., Venugopala, K. N., Chopra, D. & Guru Row, T. N. (2011). *CrystEngComm*, **13**, 591–605.

[bb8] Nayak, S. K., Venugopala, K. N., Chopra, D., Vasu & Guru Row, T. N. (2010). *CrystEngComm*, **12**, 1205–1216.

[bb9] Nayak, S. K., Venugopala, K. N., Govender, T., Kruger, H. G., Maguire, G. E. M. & Row, T. N. G. (2011). *Acta Cryst.* E**67**, o3069–o3070. 10.1107/S1600536811043649 PMC3247460 22220078

[bb10] Sheldrick, G. M. (2008). *Acta Cryst.* A**64**, 112–122. 10.1107/S0108767307043930 18156677

[bb11] Spek, A. L. (2009). *Acta Cryst.* D**65**, 148–155. 10.1107/S090744490804362X PMC2631630 19171970

